# Advanced Exergy-Based Optimization of a Polygeneration System with CO_2_ as Working Fluid

**DOI:** 10.3390/e26100886

**Published:** 2024-10-21

**Authors:** Jing Luo, Qianxin Zhu, Tatiana Morosuk

**Affiliations:** Institute for Energy Engineering, Technische Universität Berlin, Marchstr. 18, 10587 Berlin, Germany

**Keywords:** polygeneration, carbon dioxide, supercritical cycle, advanced exergy-based analysis, optimization

## Abstract

Using polygeneration systems is one of the most cost-effective ways for energy efficiency improvement, which secures sustainable energy development and reduces environmental impacts. This paper investigates a polygeneration system powered by low- to medium-grade waste heat and using CO_2_ as a working fluid to simultaneously produce electric power, refrigeration, and heating capacities. The system is simulated in Aspen HYSYS^®^ and evaluated by applying advanced exergy-based methods. With the split of exergy destruction and investment cost into avoidable and unavoidable parts, the avoidable part reveals the real improvement potential and priority of each component. Subsequently, an exergoeconomic graphical optimization is implemented at the component level to improve the system performance further. Optimization results and an engineering solution considering technical limitations are proposed. Compared to the base case, the system exergetic efficiency was improved by 15.4% and the average product cost was reduced by 7.1%; while the engineering solution shows an increase of 11.3% in system exergetic efficiency and a decrease of 8.5% in the average product cost.

## 1. Introduction

According to the *World Energy Outlook* published by the International Energy Agency [1], energy efficiency improvement is one of the most important elements for achieving sustainable development. Polygeneration systems, which simultaneously generate three or more energy products in a single integrated process, can effectively increase the system efficiency and have a large potential for decreasing the cost of the products. Therefore, increased attention is being paid to the design and optimization of polygeneration systems [2,3].

In this paper, a polygeneration system using CO_2_ as the working fluid is optimized. The system can simultaneously produce electricity, heating, and refrigeration capacities, and it is designed to be powered by low- to medium-grade waste heat. Because of the unique thermophysical properties of CO_2_ near its critical point [4], the system is expected to be compact and thermodynamically very efficient while having a low product cost.

**Figure 1:**
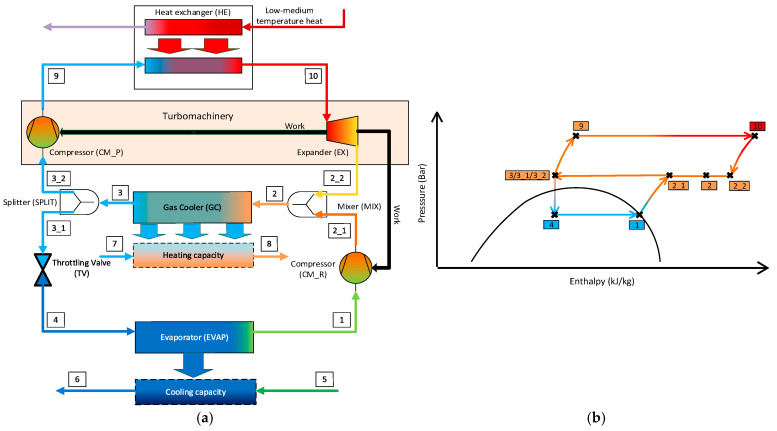
Process flow diagram (**a**) and pressure–enthalpy diagram (**b**) of the proposed polygeneration system.

Figure 1 shows a flow diagram of the evaluated polygeneration system and the corresponding thermodynamic cycle in a pressure–enthalpy diagram. The initial idea of this system design was inspired by a heat-driven compression refrigeration machine, which couples a closed-cycle gas-turbine cycle (closed direct/power cycle) with a transcritical vapor-compression refrigeration cycle (inverse cycle) via a gas cooler (GC), a mixer (MIX), and a splitter (SPLIT):

- The power cycle consists of a compressor for the power cycle (CM–P), a heat exchanger (HE), and an expander (EX). The “driving energy” is a medium-temperature heat source.
- The refrigeration cycle consists of a throttling valve (TV), an evaporator (EVAP), and a compressor for the refrigeration cycle (CM–R). The refrigeration capacity is generated within the EVAP.

The power produced by the power cycle is used to drive the refrigeration cycle.

The polygeneration system (Figure 1) has been evaluated using different methods based on the exergy-based analysis [5] and using single-objective and multi-objective optimizations [6]. In this paper, a graphical method of advanced exergy-based optimization is applied. The novelty of this research is the application of this method, for the first time, to the system consisting of the direct and reverse thermodynamic cycles.

## 2. Methodology

In this paper, the advanced exergy-based methods (reported in detail and applied for power [7] and refrigeration [8] cycles) are applied and modified for evaluating and optimizing the polygeneration system. Compared to a conventional exergetic analysis, an advanced exergy-based analysis reveals the real improvement potential of each component (the kth component) as well as the interdependencies among components [7].

### 2.1. Advanced Exergy-Based Analysis

Equations (1) and (2) present the basic idea of an advanced exergy-based analysis by dividing both the exergy destruction rate ${\dot{E}}_{D,k}$ and the associated investment cost ${\dot{Z}}_{D,k}$ of the kth component into unavoidable (superscript *UN*) and avoidable (superscript *AV*) parts to identify the thermodynamic and economic potential for improvement:

(1) $${\dot{E}}_{D,k}={\dot{E}}_{D,k}^{UN}+{\dot{E}}_{D,k}^{AV}$$

(2) $${\dot{Z}}_{D,k}={\dot{Z}}_{k}^{UN}+{\dot{Z}}_{k}^{AV}$$

The unavoidable part of the exergy destruction (${\dot{E}}_{D,k}^{UN}$ and the corresponding cost of the unavoidable part of the exergy destruction ${\dot{C}}_{D,k}^{UN}$) cannot be reduced because of the availability and cost of materials, manufacturing methods, and other technological limitations. The unavoidable investment cost (${\dot{Z}}_{D,k}^{UN}$) for each system component can be calculated by assessing the minimum values of ${\left(\frac{{\dot{Z}}_{k}}{{\dot{E}}_{P.k}}\right)}^{UN}$ [7].

In Table 1, the definitions of fuel and product for each component and for the overall system are given. For the compressor (CM_R) and the throttling valve (TV), the separate consideration of the thermal (superscript *T*) and mechanical (superscript *M*) parts of the physical exergy is required [9]. The advanced exergy-based analysis is initially conducted for a workable design called “base case”. Then, a “best case” as well as a “worst case” are assumed for the kth component to compute its unavoidable exergy destruction ${\dot{E}}_{D,k}^{UN}$ and the unavoidable investment cost ${\dot{Z}}_{k}^{UN}$, respectively. The parameters selected for these three cases are listed in Table 2. Moreover, the “overall-system approach” [8] is applied for calculating the unavoidable parts of each component by simulating the entire system with all selected parameter values for that corresponding case only once, which has the advantage of less computation time compared to the “component approach” [7] that needs to simulate each component separately for the “best” and “worst” cases.

Detailed economic and conventional exergoeconomic analyses for the poligeneration system are reported by the authors in [4,5].

In addition, a modified exergetic efficiency $\varepsilon_{k}^{AV}$ and a modified exergoeconomic factor $f_{k}^{AV}$ are computed by Equations (3) and (4), respectively. These indicators provide design engineers with more information for the further evaluation and improvement of the system at the component level [7].

(3) $$\varepsilon_{k}^{AV}=\frac{{\dot{E}}_{P,k}}{{\dot{E}}_{F,k}-{\dot{E}}_{D,k}^{UN}}=1-\frac{{\dot{E}}_{D,k}^{AV}}{{\dot{E}}_{F,k}-{\dot{E}}_{D,k}^{UN}}$$

(4) $$f_{k}^{AV}=\frac{{\dot{Z}}_{k}^{AV}}{{\dot{Z}}_{k}^{AV}+{\dot{C}}_{D,k}^{AV}}=\frac{{\dot{Z}}_{k}^{AV}}{{\dot{Z}}_{k}^{AV}+c_{F,k}{\dot{E}}_{D,k}^{AV}}$$

### 2.2. Advanced Exergy-Based Graphical Optimization

**Figure 2:**
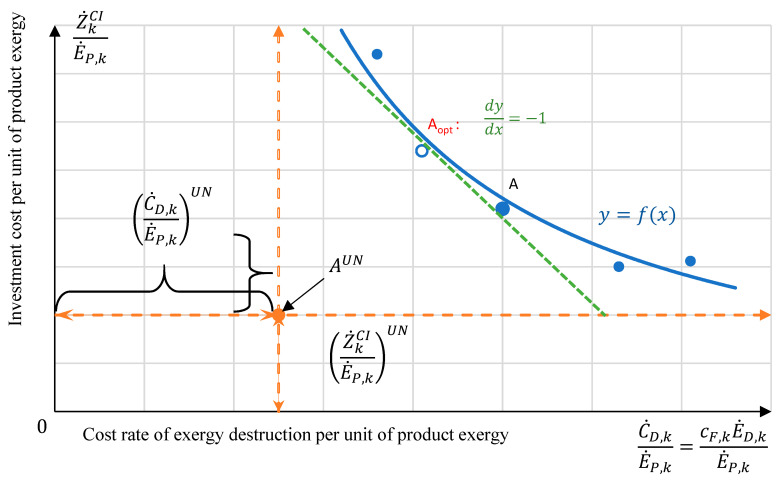
Advanced exergy-based graphical optimization by minimizing the sum of the associated investment cost rate and the exergy destruction cost rate for the kth component, adapted from [7].

As discussed in [7], the relation of $\frac{{\dot{Z}}_{k}^{CI}}{{\dot{E}}_{P,k}}$ and $\frac{{\dot{C}}_{D,k}}{{\dot{E}}_{P,k}}$ of the kth component could be presented by a curve having a horizontal and a vertical asymptote (Figure 2). The horizontal asymptote indicates the unavoidable investment cost rate per unit of product exergy ${\left(\frac{{\dot{Z}}_{k}^{CI}}{{\dot{E}}_{P,k}}\right)}^{UN}$ calculated with the parameters of the component corresponding to the “worst case”, while the vertical asymptote represents the cost rate associated with the unavoidable exergy destruction within the component per unit of product exergy ${\left(\frac{{\dot{C}}_{D,k}}{{\dot{E}}_{P,k}}\right)}^{UN}$ calculated with the parameters given for the “best case”. The optimal design point of the component can be found at the point where the derivative of the curve y = f(x) shown in Figure 2 equals to −1, $\frac{dy}{dx}$ = −1.

**Figure 3:**
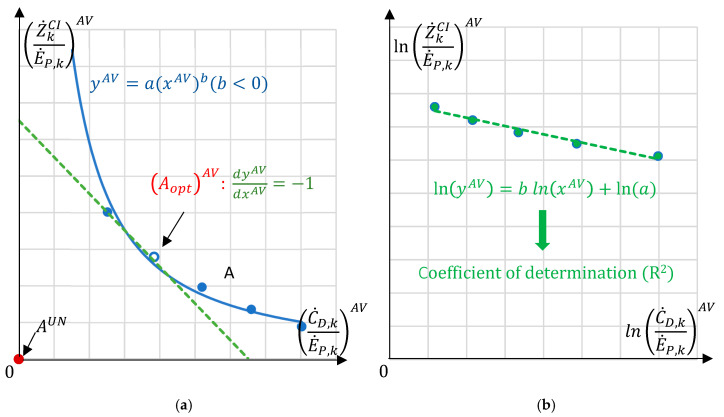
Modified graphical optimization: (**a**) minimizing the cost objective with the newly set zero point; (**b**) linearization of the fitted curve along with the least squares regression line.

In this work, the aforementioned optimization is slightly modified by calculating the intersection point A^UN^ of two asymptotes; then, this is set as the new zero point of a modified x-y diagram of ${\left(\frac{{\dot{C}}_{D,k}}{{\dot{E}}_{P,k}}\right)}^{AV}$ to ${\left(\frac{{\dot{Z}}_{k}^{CI}}{{\dot{E}}_{P,k}}\right)}^{AV}$, as shown in Figure 3a. In this newly modified diagram, the fitted function can be expressed as y = ax^b^ with b < 0. In Figure 3b, the fitted function is linearized and simplified by taking the logarithms of both sides. The easier the function is, the simpler the process of curve fitting is. Now, with the linear function ln⁡y=b ln⁡x+ln⁡ a, the problem can be defined as a linear regression problem, and the goodness-of-fit can be shown by the coefficient of determination *R*^2^ of the regression line [10]. An R^2^ of 1 indicates that the regression predictions fit the data perfectly. After the values of a and b are obtained from the linear curve fitting process, the optimal point ${\left(A_{opt}\right)}^{AV}$ of the new curve with the consideration of only avoidable parts, similarly, can be found by the point with $\frac{dy}{dx}$ = −1, and the unavoidable part needs to be added to the avoidable optimal results to compute the final $A_{opt}$.

## 3. Results and Discussion

In this section, the advanced exergy-based results of the proposed polygeneration system are given and discussed in detail, which include the results of advanced exergetic and exergoeconomic analyses for evaluating the component potential improvement and the optimization results based on the advanced exergy-based graphical optimization.

### 3.1. Results of Advanced Exergy-Based Analyses

In Table 3, the results obtained from the advanced exergy-based analyses are presented. The absolute value of the avoidable exergy destruction ${\dot{E}}_{D,k}^{AV}$, which reveals the real potential of improvement within each component, is in descending order of magnitude: TV, GC, HE, EX, CM_R, MIX, CM_P, and EVAP. If we compare the unavoidable to the avoidable parts of the components, the heat exchangers show the tendency of ${\dot{E}}_{D,k}^{UN}\gg {\dot{E}}_{D,k}^{AV}$; for the turbomachinery, ${\dot{E}}_{D,k}^{UN}\ll {\dot{E}}_{D,k}^{AV}$. However, regarding the $\varepsilon_{k}^{AV}$, the turbomachinery shows its highest efficiency (94.4% for the EX, 90.5% for the CM_P, and 90.3% for the CM_R), which indicates that the space available for technical modifications of the turbomachinery is rather small. The avoidable exergy destruction within the turbomachines may be caused more by the irreversibility occurring in the other components, an assumption that could be further proven by an advanced exergy-based method for splitting the exergy destruction into endogenous and exogenous parts. The endogenous and exogenous parts will be discussed in a future publication. Moreover, as the TV and MIX cannot be improved by themselves, one can conclude that the GC has the highest potential for improvement with the highest $\;{\dot{E}}_{D,k}^{AV}$ value and the HE with the second-highest ${\dot{E}}_{D,k}^{AV}$ comes next. Structural optimization needs to be carried out to improve the performance of TV and MIX further and the overall system to determine the best topology for the proposed polygeneration system.

### 3.2. Results of Advanced Exergy-Based Graphical Optimization

**Figure 4:**
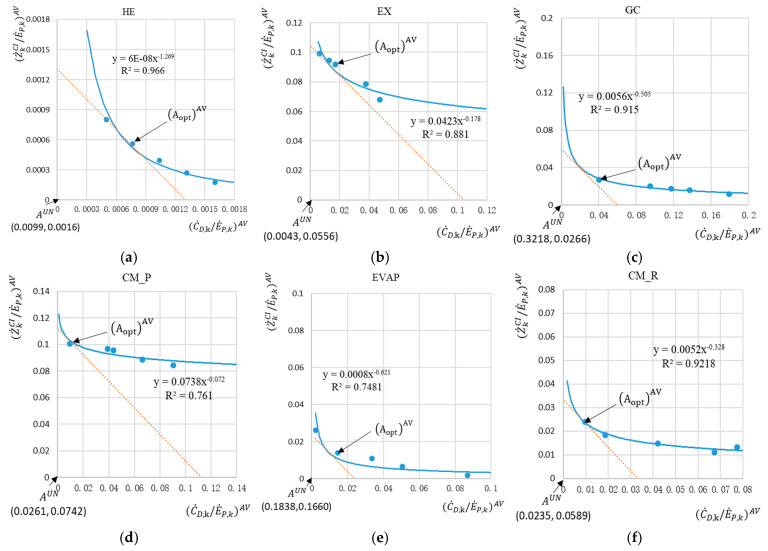
Exergoeconomic graphical component optimization based on the avoidable parts for the components of the polygeneration system: (**a**) heater (HE); (**b**) expander (EX); (**c**) gas cooler (GC); (**d**) compressor for the power cycle (CM_P); (**e**) evaporator (EVAP); and (**f**) compressor for the refrigeration cycle (CM_R).

The curve fitting of the avoidable parts, ${\left(\frac{{\dot{C}}_{D,k}}{{\dot{E}}_{P,k}}\right)}^{AV}$ to ${\left(\frac{{\dot{Z}}_{k}^{CI}}{{\dot{E}}_{P,k}}\right)}^{AV}$, for each component with its fitted function (y = ax^b^ with b < 0) is illustrated in Figure 4. In addition, the coefficient of determination R^2^ is also calculated for each curve to show how well the curve fits the original simulation data. The curves for the components HE, GC, and CM_R all fitted well with their R^2^ values being above 0.9, while the curves for the CM_P and EVAP have relatively poor values of R^2^, which may, to some extent, affect the identification of their optimal points.

Table 4 summarizes the optimal results obtained for each component based on the graphical optimization. The GC requires an improvement of its pinch point temperature difference from 5 K to 2 K, and the pinch point temperature difference for HE, similarly, needs to be reduced to 15 K from its initial setting of 20 K in the base case. For the EX, the optimal value of $\eta_{EX}$ remains the same, which can be confirmed by the limited capabilities for improving this component with its high value for the exergetic efficiency based on avoidable values:$\;\varepsilon_{EX}^{AV}=94.4\%$. The components CM_P and CM_R require an improvement in their isentropic efficiencies: from 85% in the base case to $\eta_{CM\_P}=90\%$ and $\eta_{CM\_R}=92\%$, respectively.

However, all optimal parametric values calculated for each component could only be considered by design engineers as theoretical optimization results. Engineers should also consider the current technical development and the additional costs of implementing the combination of these optimal values in the real design. Thus, an engineering solution for this system is also presented in Table 4 based on the current economic and technical background. In the engineering solution scenario, no modifications are required for the two compressors based on their current high values of $\varepsilon^{AV}$ over 90%; otherwise, a further improvement will result in a high penalty associated with the purchased equipment cost. On the contrary, modifications of the minimum temperature differences in heat exchangers are relatively less costly and easier to achieve. However, we should mention that it may be difficult to operate the GC with a ${\Delta T}_{GC}\;$= 2 K, which requires special materials and techniques.

Compared to the base case, the exergetic efficiency for the overall system ($\varepsilon_{Overall}$) increases by 15.4% and 11.3% for the cases with optimization results and with the engineering solution, respectively. Simultaneously, the overall average product cost decreases by 7.1% for the optimization results case and by 8.5% for the engineering solution case. Thus, a “cost optimum” is obtained by the so-called engineering solution. From these results, we conclude that optimizing single components in isolation does not, in general, lead to the system optimal design and that the design engineers must critically review the results of any theoretical optimization before implementation.

**Figure 5:**
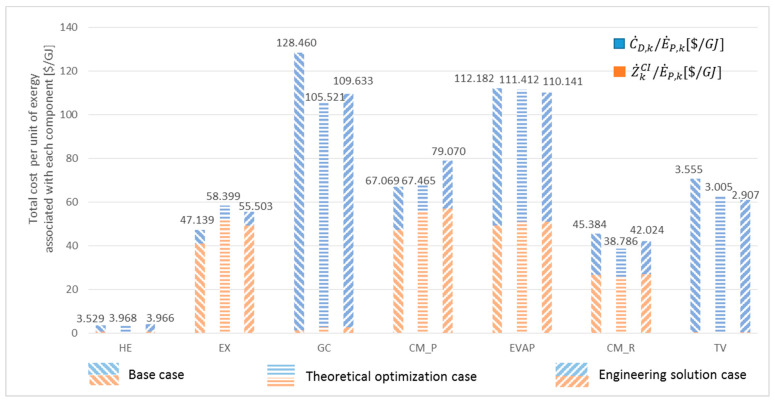
Total cost $\left(\frac{{{\dot{Z}}_{k}^{C\dot{I}}+C}_{D,k}}{{\dot{E}}_{P,k}}\right)$ associated with each system component (USD/GJ).

Figure 5 shows the distribution of the total cost $\left(\frac{{\dot{Z}}_{k}^{C\dot{I}}+C_{D,k}}{{\dot{E}}_{P,k}}\right)$ associated with each system component for the base case and the optimizations. The most significant difference can be observed for the GC.

## 4. Conclusions

In this work, advanced exergy-based analyses and optimizations were conducted for the polygeneration system using CO_2_ as the working fluid at the assumption of a refrigeration capacity of 100 kW with heat recovery of the generation of hot water at the temperature of 65 °C. By applying advanced exergy-based analyses, the avoidable inefficiencies within the system components were identified. This information assists designers in further improving the system performance from the thermodynamic and cost viewpoints. Conventional exergetic analysis [5,6] showed that the improvement priorities for the components in the overall system should be in the order of the heater (HE), the gas cooler (GC), the throttling valve (TV), the compressor for the refrigeration cycle (CM_R), the expander (EX), the evaporator (EVAP) and the compressor for the power cycle (CM_P). However, the advanced exergetic analysis suggested that the priority of technical modification for the components should be given to the gas cooler (GC), followed by the heater (HE) as the throttling process of the throttling valve (TV) with the highest avoidable exergy destruction value could not be improved by itself. A total amount of 45.2 kW, 30.8% of the overall exergy destruction rate, could be lowered with the consideration of only the overall system avoidable part (calculated by setting all parametric variables in the “best” condition with maximum efficiency for the system).

An exergoeconomic graphical optimization focusing only on the avoidable parts of components was carried out. The optimization results revealed an improvement in terms of system exergetic efficiency by more than 15%, with a reduction of more than 7% in the average product cost. However, no interactions among components were included in the advanced exergoeconomic analysis. Thus, these “optimization” results can be further improved. This fact is demonstrated by the results of the engineering solution presented here. It should be noted, however, that single-component optimization is an easily implemented and practical approach for improving the system performance with less computation time; it is especially user-friendly for non-programmers [10].

However, there is still potential for improvement. As indicated through the exergetic analysis, the optimization results, which might require further modification of the structure, especially the throttling valve (TV), need to be further investigated. Moreover, for the turbomachine (EX and CM_P), which showed an increase in the overage total cost in the optimization results case, sensitivity analyses regarding the turbine inlet temperature and turbine inlet pressure might also be necessary for further research.

## Figures and Tables

**Table 1:** Definition of the exergetic fuel and product for each component and the overall system.

Component	${\dot{E}}_{F}$	${\dot{E}}_{P}$
HE	${\dot{E}}_{11}-{\dot{E}}_{12}$	${\dot{E}}_{10}-{\dot{E}}_{9}$
EX	${\dot{E}}_{10}-{\dot{E}}_{2\_2}$	${\dot{W}}_{EX}$
GC	${\dot{E}}_{2}-{\dot{E}}_{3}$	${\dot{E}}_{8}-{\dot{E}}_{7}$
CM_P	${\dot{W}}_{CM\_P}$	${\dot{E}}_{9}-{\dot{E}}_{3\_2}$
CM_R	${\dot{W}}_{CM\_R}+{\dot{E}}_{1}^{T}$	${\dot{E}}_{2\_1}^{M}-{\dot{E}}_{1}^{M}+{\dot{E}}_{2\_1}^{T}$
EVAP	${\dot{E}}_{4}-{\dot{E}}_{1}$	${\dot{E}}_{6}-{\dot{E}}_{5}$
TV	${\dot{E}}_{3\_1}^{M}-{\dot{E}}_{4}^{M}+{\dot{E}}_{3\_1}^{T}$	${\dot{E}}_{4}^{T}$
MIX	Dissipative Component: ${\dot{E}}_{D}={\dot{E}}_{2\_1}+{\dot{E}}_{2\_2}-{\dot{E}}_{2}$	
SPLIT	-	-
Overall System	${\dot{E}}_{11}-{\dot{E}}_{12}$	${\dot{(E}}_{6}-{\dot{E}}_{5})+{\dot{W}}_{net}+{\dot{(E}}_{8}-{\dot{E}}_{7})$

**Table 2:** Values of parameters assumed for the splitting of exergy destructions and investment costs into avoidable/unavoidable parts.

Component	Parameter [Unit]	“Best” Case	Base Case	“Worst” Case
HE	${\Delta T}_{HE}[K]$	5	20	40
EX	$\eta_{EX}[\%]$	98	90	70
GC	${\Delta T}_{GC\;}[K]$	1	5	10
CM_P	$\eta_{CM_{P}}[\%]$	95	85	70
EVAP	${\Delta T}_{EVAP}[K]$	1	5	10
CM_R	$\eta_{CM\_R}[-]$	95	85	70

**Table 3:** Results of advanced exergetic and exergoeconomic analyses for the base case.

Component	${\dot{E}}_{D,k}^{UN}\left[kW\right]$	${\dot{E}}_{D,k}^{AV}\left[kW\right]$	$\varepsilon_{k}^{AV}[\%]$	${\left({\dot{Z}}_{k}^{CI}\right)}^{AV}[\$/h]$	$f_{k}^{AV}[\%]$
HE	62.21	5.27	95.5	0.044	62.3
EX	1.02	4.49	94.4	6.967	95.0
GC	20.60	6.31	77.9	0.394	38.3
CM_P	1.12	2.56	90.5	2.333	87.7
EVAP	2.78	1.06	86.8	0.075	39.5
CM_R	1.93	4.45	90.3	0.851	60.1
TV	13.93	8.73	61.5	0.002	0.5
MIX	9.58	2.87	-	0.000	0.0

**Table 4:** Results for the overall system in the base case, optimal case, and engineering solution case.

	Base Case	Optimization Results	Engineering Solution	Improvement Potential
**Operating parameters for each component**				
HE	Δ*T* = 20 K	Δ*T* = 15 K	Δ*T* = 15 K	high
EX	*η* = 0.9	*η* = 0.9	*η* = 0.9	relatively low
GC	Δ*T* = 5 K	Δ*T* = 2 K	Δ*T* = 2 K	highest (possible but difficult)
CM_P	*η* = 0.85	*η* = 0.90	*η* = 0.85	relatively low
EVAP	Δ*T* = 5 K	Δ*T* = 4 K	Δ*T* = 4 K	lowest
CM_R	*η* = 0.85	*η* = 0.92	*η* = 0.85	low
**Exergetic and exergoeconomic analysis**				
$\;\varepsilon_{Overall}(\%)$	16.5	19.1	18.4	
${\dot{W}}_{Electricity}[kW]$	0.00	0.00	0.00	
${\dot{Q}}_{Cooling}[kW]$	6.95	7.14	7.14	
${\dot{Q}}_{Heating}[kW]$	22.21	17.21	18.21	
$c_{P,Electricity}[\$/kWh]$	0.47	0.31	0.31	
$c_{P,Cooling}[\$/kWh]$	0.82	0.57	0.58	
$c_{P,Heating}[\$/GJ]$	0.84	0.86	0.84	
$c_{P,Overall}[\$/GJ]$	0.83	0.78	0.76	
**Relative change to the base case**				
Overall exergetic efficiency		15.4%	11.3%	
Overall average product cost		−7.1%	−8.5%	

## Data Availability

Data is contained within the article.

## Nomenclature

$c_{F,k}$	fuel cost per unit of exergy of *k*th component, USD/kWh
$c_{P,k}$	product cost per unit of exergy of *k*th component, USD/kWh
${\dot{C}}_{D,k}$	destruction cost rate associated with *k*th component, USD/h
${\dot{E}}_{i}^{M}$	mechanical exergy rate of *i*th stream, kW
${\dot{E}}_{i}^{T}$	thermal exergy rate of *i*th stream, kW
${\dot{E}}_{F,k}$	fuel exergy rate of *k*th component, kW
${\dot{E}}_{P,k}$	product exergy rate of *k*th component, kW,
${\dot{E}}_{D,k}$	exergy destruction rate of *k*th component, kW
${\dot{E}}_{D,k}^{AV}$	avoidable exergy destruction rate of *k*th component, kW
${\dot{E}}_{D,k}^{UN}$	unavoidable exergy destruction rate of *k*th component, kW
$f_{m}$	material factor for the calculation of purchased equipment cost, -
$f_{k}$	exergoeconomic factor of *k*th component, %
$f_{k}^{AV}$	modified exergoeconomic factor of *k*th component, %
*T_HS_*	thermodynamic average temperature of the stream of matter providing the low to medium-grade heat, K
${\dot{Z}}_{k}^{CI}$	capital investment cost rate of *k*th component, USD/h
${\dot{Z}}_{k}^{AV}$	avoidable capital investment cost rate of *k*th component, USD/h
${\dot{Z}}_{k}^{UN}$	unavoidable capital investment cost rate of *k*th component, USD/h
ΔT	pinch point temperature difference, K
$\varepsilon_{k}$	exergetic efficiency of *k*th component, %
$\varepsilon_{k}^{AV}$	modified exergetic efficiency of *k*th component, %
η	isentropic efficiency, %
**Abbreviations**	
CM–P	compressor in power sub-cycle
CM–R	compressor in refrigeration sub-cycle
EVAP	evaporator
EX	expander
GC	gas cooler
HE	heat exchanger
MIX	mixer
SPLIT	splitter
TV	throttling valve
